# Thermonucleases Contribute to *Staphylococcus aureus* Biofilm Formation in Implant-Associated Infections–A Redundant and Complementary Story

**DOI:** 10.3389/fmicb.2021.687888

**Published:** 2021-06-24

**Authors:** Jinlong Yu, Feng Jiang, Feiyang Zhang, Musha Hamushan, Jiafei Du, Yanjie Mao, Qiaojie Wang, Pei Han, Jin Tang, Hao Shen

**Affiliations:** ^1^Department of Orthopedics, Shanghai Jiao Tong University Affiliated Sixth People’s Hospital, Shanghai, China; ^2^Department of Clinical Laboratory, Shanghai Jiao Tong University Affiliated Sixth People’s Hospital, Shanghai, China; ^3^Department of Orthopedics, Jinjiang Municipal Hospital, Fujian, China

**Keywords:** *Staphylococcus aureus*, biofilm, thermonuclease, implant associated infections, periprosthetic joint infection

## Abstract

Biofilms formed by *Staphylococcus aureus* are one of the predominant causes of implant-associated infections (IAIs). Previous studies have found that *S. aureus* nucleases *nuc1* and *nuc2* modulate biofilm formation. In this study, we found low *nuc1*/*nuc2* expression and high biofilm-forming ability among IAI isolates. Furthermore, in a mouse model of exogenous IAIs, Δ*nuc1/2* exhibited higher bacterial load on the surface of the implant than that exhibited by the other groups (WT, Δ*nuc1*, and Δ*nuc2*). Survival analysis of the hematogenous IAI mouse model indicated that *nuc1* is a virulence factor related to mortality. We then detected the influence of *nuc1* and *nuc2* on biofilm formation and immune evasion *in vitro*. Observation of *in vitro* biofilm structures with scanning electron microscopy and evaluation of bacterial aggregation with flow cytometry revealed that both *nuc1* and *nuc2* are involved in biofilm structuring and bacterial aggregation. Unlike *nuc1*, which is reported to participate in immune evasion, *nuc2* cannot degrade neutrophil extracellular traps. Moreover, we found that *nuc1*/*nuc2* transcription is negatively correlated during *S. aureus* growth, and a possible complementary relationship has been proposed. In conclusion, *nuc1*/*nuc2* are complementary genes involved in biofilm formation in exogenous IAIs. However, *nuc2* contributes less to virulence and is not involved in immune evasion.

## Introduction

Orthopedic implants are mainly used for bone fixation and joint replacement. Owing to locally compromised host defense, implanted foreign structures are highly susceptible to microbial colonization (Zimmerli and Moser, 2012; Zimmerli, 2014). As a devastating complication after arthroplasty or internal fixation, implant-associated infections (IAIs) frequently lead to the failure of the prosthetic device or requirement of implant replacement and are associated with substantial patient morbidity (Kapadia et al., 2016; Depypere et al., 2020). Orthopedic IAIs are often caused by *Staphylococcus aureus*, although many other pathogens can lead to such infections (Arciola et al., 2005; Pulido et al., 2008). IAIs can be classified as exogenous or hematogenous (Zimmerli, 2014; Wang et al., 2017; Arciola et al., 2018). Exogenous infections, which are the most common type, occur as a consequence of direct seeding from external contaminants or contiguous spread during the perioperative period. Hematogenous infections involve bacterial seeding on implants through the bloodstream. Although hematogenous infections occur less frequently, they represent up to 20% of prosthetic joint infections (PJIs) (Sendi et al., 2011; Konigsberg et al., 2014; Tande et al., 2016).

In contrast to other infections such as bacteremia and skin abscess, microbes in IAIs generally form biofilms, which are aggregated structured bacterial communities encased in an extracellular matrix. Biofilms are responsible for the recalcitrance of implant infection to therapy and serve as a source of bacterial dissemination (Arciola et al., 2018). Biofilms are characterized by the production of extracellular polymeric substances (EPSs), which commonly comprise lipids, extracellular proteins, extracellular DNA (eDNA), and exopolysaccharides (Hobley et al., 2015; Schilcher and Horswill, 2020). EPSs typically account for 90% or more of the biofilm dry weight and perform various functions for the inhabitants, such as providing structural rigidity or protecting them from external environmental stress (Flemming and Wingender, 2010; Flemming, 2016). Researchers found that methicillin-sensitive *S. aureus* (MSSA) strains commonly produce polysaccharide intercellular adhesin (PIA)-dependent biofilms. In contrast, the release of eDNA and cell surface expression of a number of sortase-anchored proteins have been implicated in the biofilm phenotype of methicillin-resistant *S. aureus* (MRSA) (Mccarthy et al., 2015).

Extracellular DNA has been recognized as a component of the EPS matrix for a long time. However, its role in the EPS was underestimated until the discovery that it is an essential component in *Pseudomonas aeruginosa* biofilms (Whitchurch et al., 2002). Further investigation revealed that eDNA stabilizes the biofilm matrix and promotes antimicrobial resistance (Hall-Stoodley et al., 2012). In addition, two clinical studies have recently reported a relationship between the presence of eDNA in the biofilm and the outcome of orthopedic IAIs (Zatorska et al., 2017, 2018).

Extracellular DNA is released through bacterial autolysis and digested by nucleases (Okshevsky et al., 2015). According to previous reports, *S. aureus* secretes thermonuclease enzymes to regulate biofilm formation by modulating eDNA (Kiedrowski et al., 2011; Tang et al., 2011). To our knowledge, the chromosome of *S. aureus* encodes two thermonucleases, *nuc1* and *nuc2* (Tang et al., 2008; Hu et al., 2012). *nuc1*, also called micrococcal nuclease, was the first documented thermonuclease, and it is a secreted virulence factor controlled by the *SaeRS* two-component system (Olson et al., 2013). *nuc2* is a cell surface-binding protein with functional nuclease activity (Kiedrowski et al., 2014). Previous studies have reported that *S. aureus* secretes *nuc1* to degrade neutrophil extracellular traps (NETs) and kill phagocytes (Berends et al., 2010; Thammavongsa et al., 2013; Sultan et al., 2019). Interestingly, the two abovementioned phenotypes (biofilm formation and immune evasion) seem incompatible because *nuc1* upregulation contributes to immune evasion, whereas *nuc1* downregulation leads to biofilm formation. The mechanism by which nucleases regulate the survival of *S. aureus* in the IAI microenvironment remains unknown. In addition, the contribution of *nuc2* to *S. aureus* pathogenesis in biofilm-related infections and whether *nuc2* contributes to immune evasion are particularly unclear because this nuclease was more recently discovered than *nuc1* and has received limited attention.

In this study, we evaluated the activity of thermonucleases in IAI isolates. We also examined the impact of *nuc1* and *nuc2* on biofilm formation and immune evasion under *in vitro* and *in vivo* conditions. Finally, we discussed the relationship between the two nucleases and their function in *S. aureus* survival and adaptation in the IAI microenvironment.

## Results

### Low Thermonuclease Expression and High Biofilm-Forming Ability in IAI Strains

**FIGURE 1:**
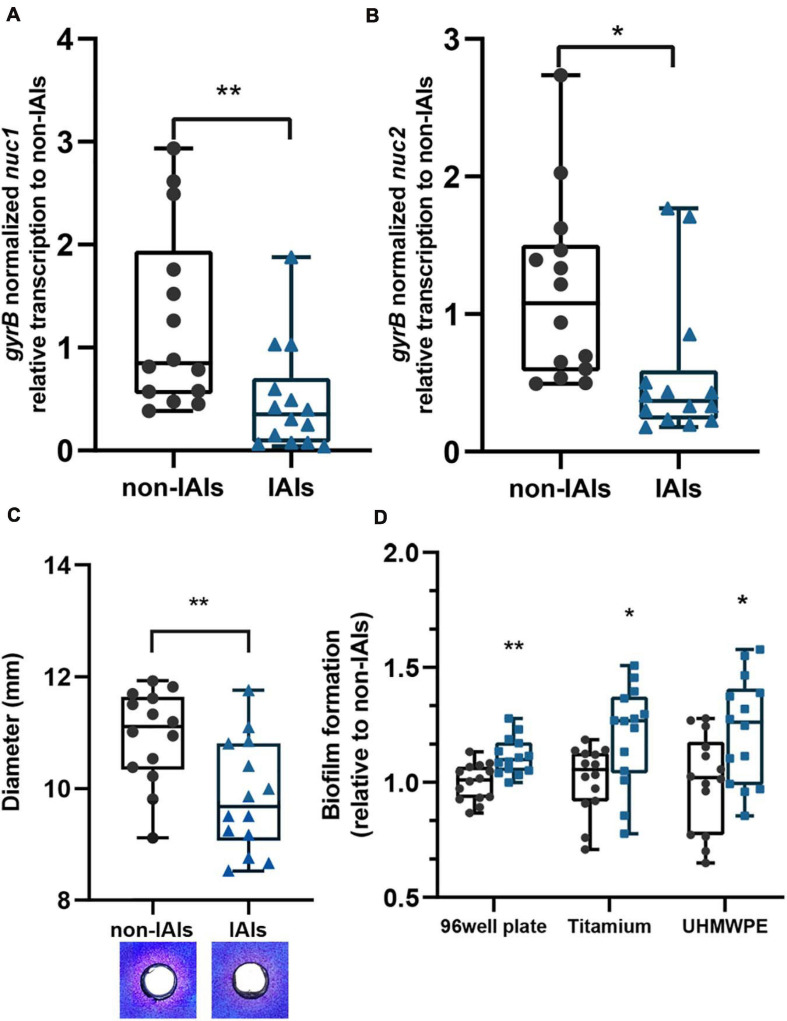
The expression levels of thermonucleases and biofilm-forming ability in clinical isolates. Expression of *nuc1* **(A)** and *nuc2* **(B)** in IAI and non-IAI isolates (*n* = 14/group) determined by qPCR. **(C)** Nuclease activity in toluidine blue DNA agar represented in the diameter of red zones. Representative images are also presented. Red zones indicate nuclease activity. **(D)** Biofilm formation of IAI (*n* = 14) and non-IAI isolates (*n* = 14) on different materials including polypropylene 96-well plates (non-IAIs = 1.334 ± 0.103), titanium disk (non-IAIs = 1.664 ± 0.229), and UHMWPE (non-IAI = 1.407 ± 0.284). Biofilm biomass was stained with crystal violet. Statistical significance was calculated using two-tail Student’s *t*-test in panels **(A**–**C)**; the multiple *t*-test (Bonferroni–Dunn’s test) was used in panel **(D)**. *p < 0.05; **p < 0.01 vs. non-IAI strains.

We analyzed the transcription levels of *S. aureus* thermonucleases among 28 clinical isolates using quantitative PCR (qPCR; Figures 1A,B). Significantly lower transcription levels of both *nuc1* (*p* < 0.01) and *nuc2* (*p* < 0.05) were observed in IAI strains (*n* = 14) than in non-IAI strains (*n* = 14). *nuc1* and *nuc2* transcription levels in the IAI group were 2.57- and 2.47-fold lower than those in the non-IAI groups, respectively. Since gene expression is highly dependent on the involved environment, we included human synovial fluid to mimic the environment encountered by the bacteria in the host, and the results were similar to isolates grown in TSB (Supplementary Figure 1).

To determine whether there were differences in nuclease enzyme activity between the two groups, thermonuclease activity was measured directly using toluidine blue DNA agar, and the zones of clearing were measured. The majority of strains from the IAI group had smaller zones of clearing than those of the strains from the non-IAI group (*p* < 0.01), which indicated lower thermonuclease activity. Representative images for each group are shown in Figure 1C.

Previous reports demonstrated that *S. aureus* nuclease (*nuc1*) could affect biofilm formation by modulating eDNA (Kiedrowski et al., 2011). Here, we noted an increased biofilm eDNA in the IAI group (Supplementary Figure 2A). By relating the eDNA amount to *nuc1* and *nuc2* expression levels, we found a moderate correlation between *nuc1* and eDNA (Pearson *R* = −0.4592; Supplementary Figure 2B), but no significant correlation was found between *nuc2* and eDNA (Pearson *R* = −0.2983, *p* > 0.05). Then, we wonder if the IAI isolates with low thermonuclease activity also have higher biofilm-forming ability. Static microtiter biofilm assay found that IAI isolates had higher biofilm-forming ability (Figure 1D). Considering that the most used materials in orthopedic implants are titanium alloy and ultra-high-molecular-weight polyethene (UHMWPE), we further performed biofilm formation assay on titanium disk and UHMWPE, and similar results were obtained (Figure 1D). These data together showed that IAI strains are more prone to form biofilms on the surface of various materials than their non-IAI counterparts.

### Construction and Characterization of *nuc1/nuc2* Mutant Strains

To study the pathogenesis of *S. aureus nuc1* and *nuc2* in IAIs, we constructed *nuc1* and/or *nuc2* mutants using the clinical IAI isolate ST1792, which we termed Δ*nuc1*, Δ*nuc2*, and Δ*nuc1/2*. After in-frame mutation, the strain genotypes were validated by Sanger sequencing (Supplementary Figure 3A). Interestingly, the colony formed by Δ*nuc1/2* was much stickier (Supplementary Figure 3B) than that formed by wild type (WT), Δ*nuc1*, and Δ*nuc2*. The same phenomenon was also observed in the USA300 *nuc1*/*nuc2* isogenic mutant (BD1281).

**FIGURE 2:**
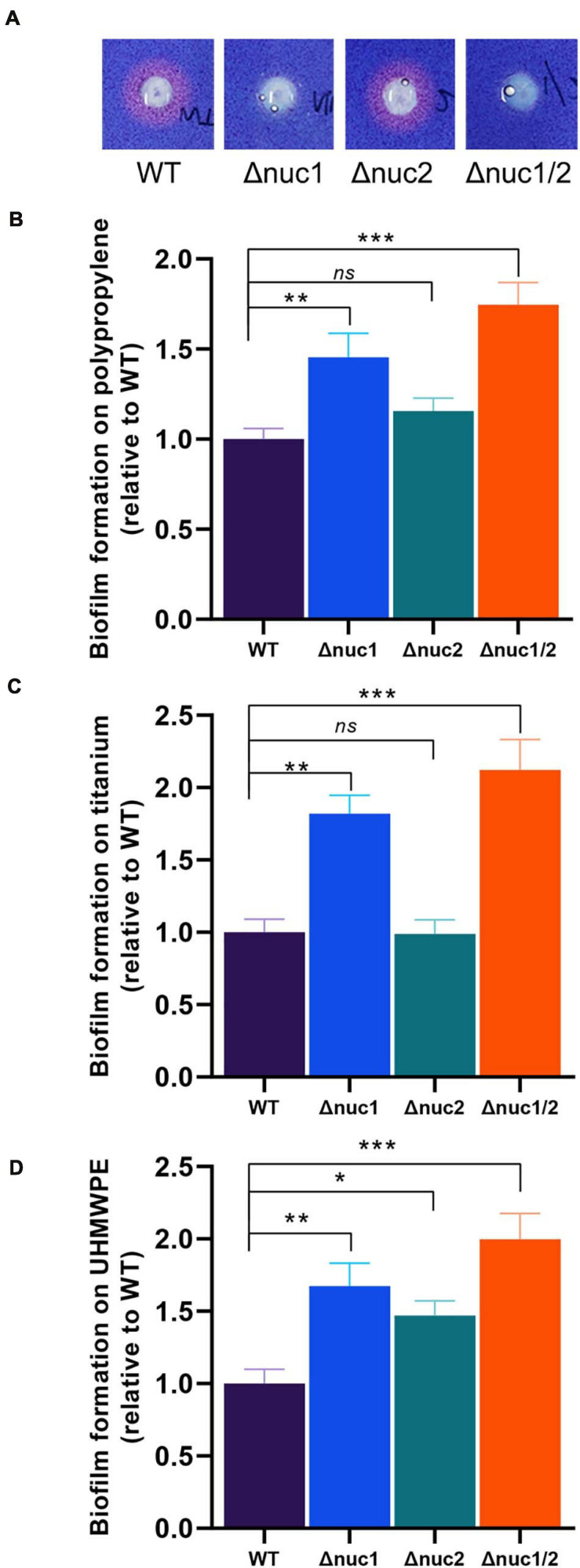
*In vitro* nuclease activity and biofilm-forming ability of ST1792 and its isogenic mutant strains. **(A)** Toluidine blue DNA agar test with red zones representing nuclease activity. **(B–D)** Biofilm-forming ability of tested strains on polypropylene 96-well plates (**B**, WT = 1.135 ± 0.055), titanium disk (**C**, WT = 1.237 ± 0.091), and UHMWPE disk (**D**, WT = 1.457 ± 0.119). Biofilm biomass was stained with crystal violet. Statistical significance was calculated using ANOVA with Dunnett multiple column comparisons. *n* = 3/group for each experiment. *p < 0.05; **p < 0.01; ***p < 0.001 vs. WT.

Nuclease activity was then compared among the four strains (Δ*nuc1*, Δ*nuc2*, Δ*nuc1/2*, and WT) using toluidine blue DNA agar (Figure 2A). No observable difference was found between Δ*nuc2* and WT with a wide area of the clearing zone. In contrast, no detectable nuclease activity was observed for Δ*nuc1* and Δ*nuc1/2*. We also quantified the biofilm-forming capacity of these strains. Following crystal violet staining, we observed that the biomass of Δ*nuc1* and Δ*nuc1/2* increased significantly (Figures 2B–D, Δ*nuc1*: *p* < 0.01, Δ*nuc1/2*: *p* < 0.001) in various materials, including titanium, UHMWPE, and polypropylene 96-well plates. However, the biomass of the Δ*nuc2* biofilm varied with the materials. For example, when grown in UHMWPE, Δ*nuc2* bacteria developed a more robust biofilm than developed by the WT bacteria. However, biofilms grown on titanium disks and 96-well plates were comparable to the WT biofilms. In addition to quantifying biofilm biomass, the number of culturable cells was also assessed. The results showed that Δ*nuc1/2* biofilms contained more bacterial cells than the other genotypes (Supplementary Figure 4).

### Δ*nuc1/2* Has Higher Biofilm-Forming Capacity in the Exogenous IAI Mouse Model

**FIGURE 3:**
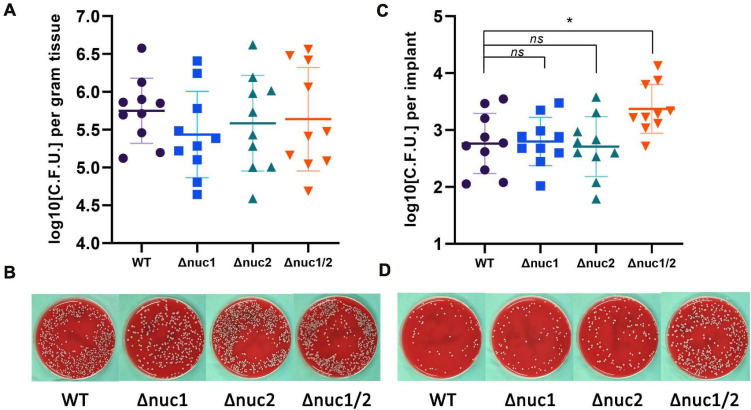
Bacterial burden in an exogenous IAI mouse model. CFU for peri-implant tissue and biofilms on implant was determined 7 days after infection. **(A)** Bacterial count for peri-implant tissues and representative photos **(B)**. **(C)** Bacterial count for biofilms on implant and representative photos **(D)**. Statistical significance was calculated using ANOVA with Dunnett multiple column comparisons. *n* = 10/group. *p < 0.05 vs. WT.

By inoculating bacteria around the implant locally, we constructed an exogenous IAI mouse model. All mice were euthanized 7 days after infection. No significant differences were found among the groups (WT, Δ*nuc1*, Δ*nuc2*, and Δ*nuc1/2*) when evaluating the bacterial load in the peri-implant tissues (Figures 3A,B). However, when quantifying adherent bacteria on the implant, a higher bacterial load was exhibited by Δ*nuc1/2* than by the WT (Figures 3C,D). Interestingly, the adherent bacterial load showed no statistical difference among the Δ*nuc1*, Δ*nuc2*, and WT groups.

**FIGURE 4:**
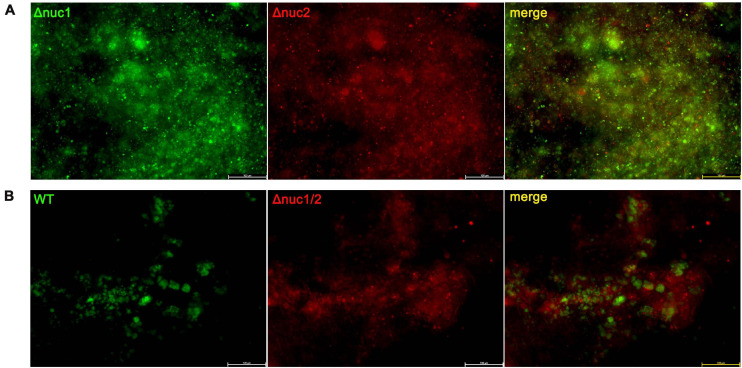
Implant from a competition infection mouse model observed with a fluorescent microscope. Implants were harvested on the seventh day since infection and observed with a fluorescence microscope. **(A)** Implant from mice infected with a ∼1:1 mixture of Δ*nuc1* (green) and Δ*nuc2* (red). **(B)** Implant from mice infected with a ∼1:1 mixture of WT (green) and Δ*nuc1/2* (red). Scale bar = 100 μm, *n* = 3/group.

To investigate the effect of nucleases on environmental adaptations *in vivo*, a competitive assay was conducted. Bacteria with different genotypes and fluorescent labels were mixed and inoculated *in vivo*. The implants were harvested on day 7 and observed under a fluorescence microscope. Groups infected with a mixture of Δ*nuc1* and Δ*nuc2* presented overlapping red and green fluorescence, and no difference was detected between them (Figure 4). The Pearson correlation test showed a strong correlation (*R* = 0.83) between Δ*nuc1* (green) and Δ*nuc2* (red) signals (Supplementary Figure 5A). However, in groups infected with a mixture of WT and Δ*nuc1/2*, a difference in bacterial distribution (Figure 4B) and a low Pearson correlation (Supplementary Figure 5B) were observed. Specifically, Δ*nuc1/2* strains labeled with mCherry exhibited broad and even distribution, whereas WT strain labeled with superfolder GFP (sfGFP) was distributed in clusters with less area covered.

### Δ*nuc1/2* Affects Bacterial Aggregation and Biofilm Structure *in vitro*

**FIGURE 5:**
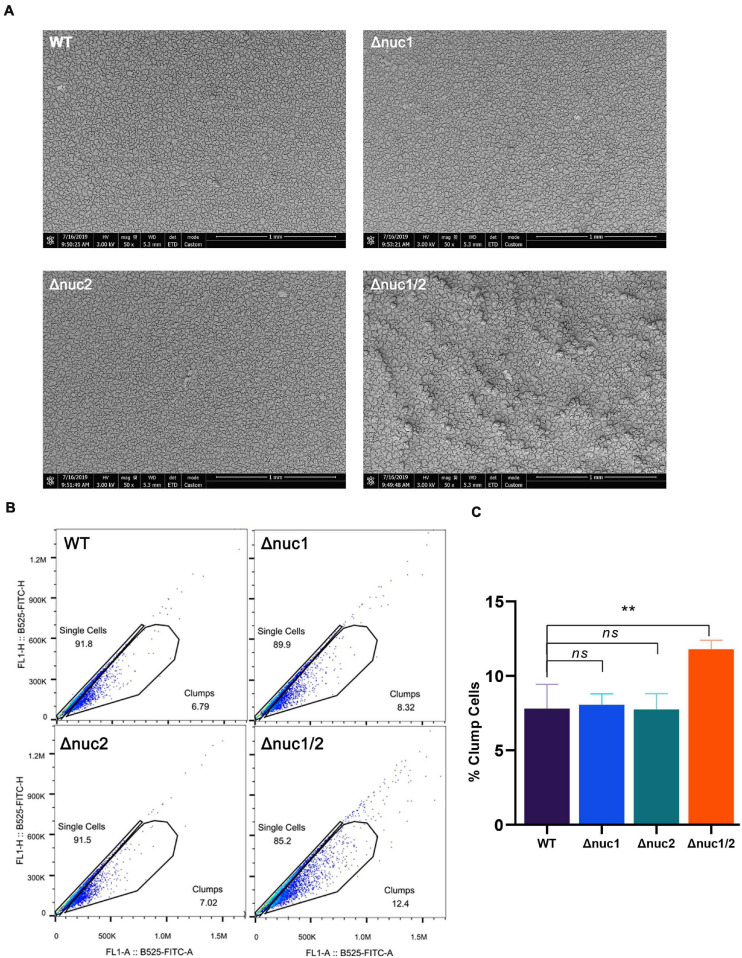
Role of *nuc1* and *nuc2* on bacterial aggregation and biofilm structure *in vitro*. **(A)** Biofilm formed by ST1792 and its isogenic mutant on titanium disk observed with SEM (scale bar = 1 mm). **(B)** Flow cytometry was used to determine bacterial aggregation. **(C)** Quantification result for the flow cytometry experiment. Statistical significance was calculated using ANOVA with Dunnett multiple column comparisons. *n* = 3/group. **p < 0.01 vs. WT.

Next, we examined biofilm structure, *in vitro*, using scanning electronic microscopy (SEM). The biofilm structure of Δ*nuc1/2* was different from that of the other three genotypes (Figure 5A). Δ*nuc1/2* bacteria developed “valley and mountain-like” structures, whereas the other bacterial strains did not. However, this difference was only observed at ×50 magnification. When the biofilm was observed at ×2,000 magnification, no difference was detected (Supplementary Figure 6). In order to observe the extracellular matrix, we used a confocal microscope. We noticed that the biofilms formed by Δ*nuc1/2* were thicker and had higher PI signals, which represent both eDNA and dead cells.

We also measured the percentage of bacterial aggregation using flow cytometry (Figures 5B,C). Δ*nuc1/2* was more likely to aggregate between bacterial cells (*p* < 0.01). No statistical difference was observed among the remaining groups (Δ*nuc1*, Δ*nuc2*, and WT).

### *nuc2* Is Not a Virulence Factor Like *nuc1* in a Hematogenous Mice Model

**FIGURE 6:**
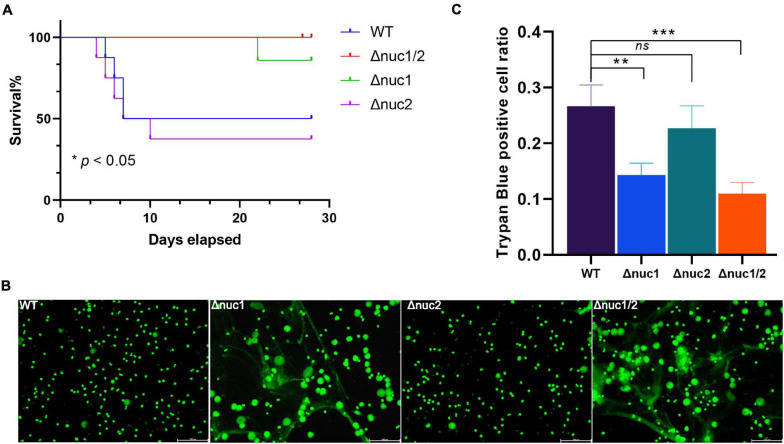
*nuc1* and *nuc2* involvement in immune evasion. **(A)** Survival curve for a hematogenous IAI mouse model infected with ST1792 WT, Δ*nuc2* (*n* = 8 each), and Δ*nuc1* and Δ*nuc1/2* (*n* = 7 each). **(B)** Representative fluorescent images for NET degradation assay. *S. aureus* were incubated with PMA-stimulated neutrophils (*n* = 3/group). A green signal represents NETs and cell nucleus, scale bar = 100 μm. **(C)** THP-1 macrophages were incubated with DNA and *S. aureus*. Cell viability was determined by trypan blue staining (*n* = 3/group). The survival curve was analyzed with a log-rank (Mantel–Cox) test. ANOVA with Dunnett multiple column comparisons was used in panel **(C)**. **p < 0.01; ***p < 0.001 vs. WT.

The work mentioned above was based on an IAI mouse model induced by surgical site contamination. However, hematogenous infections represent up to 20% of IAIs (Wang et al., 2017). Therefore, we investigated the pathogenesis of Δ*nuc1* and/or Δ*nuc2* strains in a hematogenous IAI mouse model. Based on our observations, the group infected with WT and its isogenic *nuc2* mutant had significantly higher mortality rates (*p* < 0.05, Figure 6A). Mutual comparisons of survival curves among the four groups are presented in Supplementary Figure 8. Interestingly, most of the death events occurred within 7 days of infection. However, no difference was detected in the bacterial load in peri-implant tissue and on the implant among the four groups (Supplementary Figure 9).

According to previous reports, *nuc1* is involved in immune evasion (Tang et al., 2011; Thammavongsa et al., 2013). Hence, we speculated whether *nuc2* had the same function. In the NET degradation assay, in which WT and Δ*nuc1/2* were considered positive and negative controls, respectively, we did not detect any difference when comparing Δ*nuc1* with Δ*nuc1/2* (Figure 6B). A previous study reported that *nuc1* could lead to immune cell death (Thammavongsa et al., 2013). In line with this result, the trypan blue staining assay (Figure 6C) showed increased THP-1 cell viability in the Δ*nuc1* and Δ*nuc1/2* groups when compared with the WT. However, there was no difference in cell viability between the WT and Δ*nuc2* groups or between the Δ*nuc1* and Δ*nuc1/2* groups.

### *Staphylococcus aureus* Sequentially Expresses *nuc1* and *nuc2* for Environmental Adaptation

**FIGURE 7:**
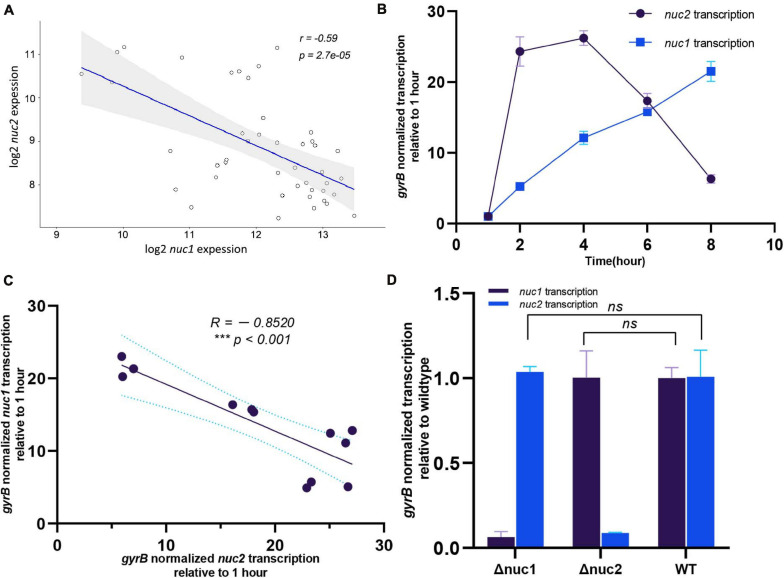
Transcription relationship between *nuc1* and *nuc2*. **(A)** *nuc1* and *nuc2* correlation found by analyzing the publicly available data GSE25454. **(B)** Transcription levels of *nuc1* and *nuc2* in WT ST1792 at various time points determined through qPCR (*n* = 3/time point). **(C)** Transcription levels of *nuc2* at various time points (2, 4, 6, and 8 h) were related to *nuc1*. **(D)** *nuc1* and *nuc2* transcription levels in ST1792 WT, Δ*nuc1*, and Δ*nuc2* determined using qPCR (*n* = 3/group). A Pearson correlation test was performed in panels **(A,C)**, and unpaired Student’s *t* test was performed in panel **(D)**.

Our study indicates that *nuc1* and *nuc2* are both essential for biofilm formation. However, the redundancy of thermonucleases in the *S. aureus* chromosome prompted us to investigate the underlying mechanism. By analyzing the public microarray dataset GSE25454, we found that *nuc1* and *nuc2* were negatively correlated (Figure 7A, *R* = −0.59, *p* < 0.001). Our qPCR results confirmed this phenomenon: during *S. aureus* growth in tryptic soy broth (TSB), *nuc2* was upregulated in 2–4 h and then decreased. In contrast, *nuc1* transcription peaked in the post-exponential stage (Figure 7B). Also, the correlation between *nuc1* and *nuc2* in our study was similar to what we found in the dataset GSE25454 (Figure 7C, *R* = −0.8520, *p* < 0.001). To exclude the possible regulation between the two genes, we also investigated *nuc1*/*nuc2* transcription in Δ*nuc2*/Δ*nuc1* (Figure 7D), and the data obtained showed no regulation between *nuc1* and *nuc2*. Such a negative correlation led to our hypothesis that *nuc1* and *nuc2* are complementary. Therefore, it is possible that *nuc2* functions in the early growth phase and that *nuc1* plays its role during the later phase.

### *Staphylococcus aureus* Modulates Nuclease Transcription When Exposed to Antibiotics

**FIGURE 8:**
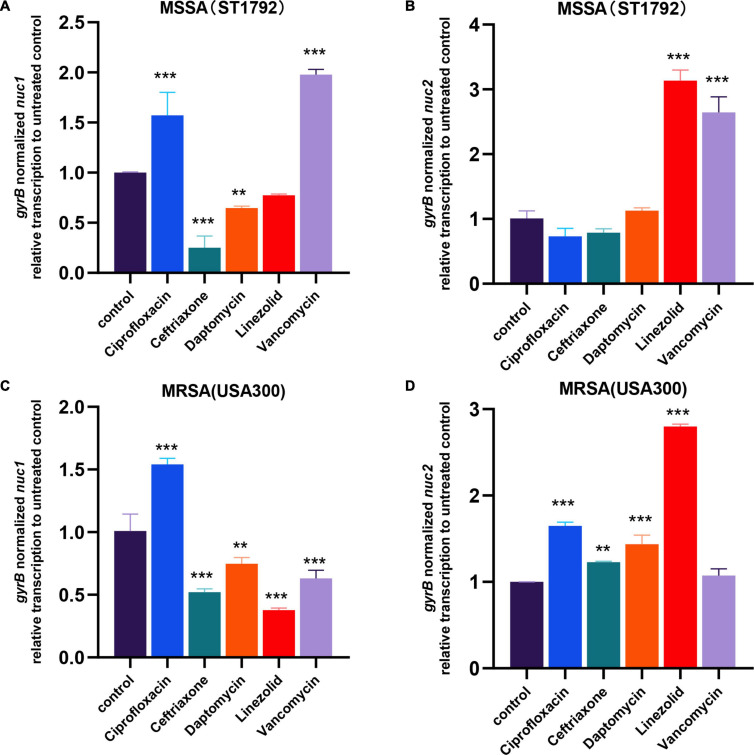
*nuc1* and *nuc2* transcription changes when exposed to antibiotics in MRSA (USA300) and MSSA (ST1792). **(A,B)** *nuc1* **(A)** and *nuc2* **(B)** transcription changes in MSSA (ST1792). **(C,D)** *nuc1* **(C)** and *nuc2* **(D)** transcriptional changes in MRSA (USA300). Statistical significance was calculated using ANOVA with Dunnett multiple column comparisons. **p < 0.01; ***p < 0.001 vs. non-treated control.

In the first part of our study, we found low expression of nucleases in IAI isolates. Considering patients with IAIs generally require long-term antibiotic administration, we further speculated whether antibiotics would affect *nuc1/2* expression. We then exposed MRSA (USA300) and MSSA (ST1792) to several of the most commonly used antibiotics at sub-minimum inhibitory concentration (MIC) levels. First, we determined the MIC and sub-MIC of both strains, and the results are listed in Table 1. Following sub-MIC exposure, qPCR was used to measure *nuc1* and *nuc2* transcriptions (Figure 8).

**TABLE 1:** Minimum Inhibitory Concentration and sub-MIC for strains tested^#^.

Strain	(μ g/ml)	Ciprofloxacin	Ceftriaxone	Daptomycin	Linezolid	Vancomycin
ST1792	MIC	0.25	2	>16	1	2
USA300	MIC	0.25	>8	>16	1	2
ST1792	sub-MIC*	0.125	1	8	0.5	1
USA300	sub-MIC*	0.125	4	8	0.5	1

#MIC level was determined based on three technical replicates. *Sub-MIC was equal to 1/2 MIC.

We found that MSSA and MRSA had different responses to antibiotics. Fewer differences in expression patterns were seen for *nuc1* in the two strains with only a major shift seen for vancomycin (decreased in MRSA but increased in MSSA). In both ST1792 and USA300, *nuc1* was upregulated when exposed to ciprofloxacin and downregulated after exposure to ceftriaxone and daptomycin. However, for *nuc2* expression, an increase was observed in both MRSA and MSSA strains after linezolid. Ciprofloxacin, ceftriaxone, and daptomycin increased *nuc2* expression only in the MRSA strain. Finally, *nuc2* expression increased in response to vancomycin for the MSSA strain, which is similar to *nuc1*.

## Discussion

To our knowledge, this is the first study reporting that *S. aureus* IAI isolates have low nuclease (*nuc1* and *nuc2*) expression levels, which may be relevant for the high biofilm-forming capacity of IAI isolates. By constructing nuclease mutant strains, we found that Δ*nuc1/2* exhibited higher biofilm-forming capacity in an exogenous IAI mouse model. However, a previous study reported that *nuc1* and *nuc2* had no significant impact on biofilm formation using a murine model of catheter-associated biofilm formation (Beenken et al., 2012). Although the reason underlying this discrepancy with our results is unclear, we noticed that the strain used is different, which could explain, in part, the discrepancies observed.

According to previous reports, *nuc1* regulates biofilm formation by modulating eDNA in the biofilm matrix (Kiedrowski et al., 2011; Tang et al., 2011). However, the impact of *nuc2* on biofilms has received limited attention. In this study, we noticed that Δ*nuc1/2* strains formed sticky colonies and distinct biofilm morphology. One possible explanation for this phenomenon is that the mutation of both *nuc1* and *nuc2* leads to the accumulation of eDNA, which in turn may increase colony and biofilm viscosity. This hypothesis is partially corroborated by a previous study (Kaito et al., 2011), where it was reported that the presence of eDNA increases extracellular matrix viscosity. Also, biofilms observed with confocal microscopy showed that Δ*nuc1/2* formed thicker biofilms with higher PI signal. According to a previous report, eDNA degradation is involved in the “exodus” and “dispersal” steps during biofilm maturation (Moormeier et al., 2014). Their study prompted us to speculate that a high eDNA content in the biofilm matrix may make both live and dead bacteria unable to egress from the biofilm and get trapped, thus resulting in a thicker biofilm with a higher PI signal. Nevertheless, we cannot exclude other possible mechanisms accounting for the observed phenotypes. Since previous studies have not determined if *nuc1* and *nuc2* have an influence on other biofilm-related genes, the detected phenomenon could also be caused by the regulation between nucleases and other genes.

By analyzing *nuc1* and *nuc2* transcription levels at different time points, we found that *nuc1* and *nuc2* transcription levels were temporally regulated during *S. aureus* growth, and this was also reported in a previous study (Hu et al., 2012). It seems *nuc2* was expressed when cell density was low, and *nuc1* was prone to be expressed at high cell density. Such temporal gene regulation was most likely dependent on a quorum-sensing system. However, *agr*, the most well-studied quorum sensing, is not directly associated with *nuc1* or *nuc2* regulation (Olson et al., 2013; Kiedrowski et al., 2014). Hence, other quorum-sensing systems (e.g., LuxS) and stimuli rather than population density might be involved in the temporal regulation of thermonucleases. Meanwhile, it should be noted that biofilms are not synchronized in terms of growth phase and that it would lead to special difference in gene expression. Previous studies found that *nuc1* and *nuc2* are highly expressed in the peripheral colony, highlighting the need to study in more detail the spatial regulation of *nuc1* and *nuc2* in biofilms (Kaito et al., 2011).

The redundancy of *nuc1* and *nuc2* does not mean equal contribution to *S. aureus* virulence. For instance, *nuc1* in hematogenous IAIs contributed to the mortality rate of infection while *nuc2* did not. Interestingly, we observed that most deaths, in the hematogenous IAI model, occurred during the first 7 days of infection. Based on the knowledge that adaptive immunity takes 4–7 days to mount a response (Iwasaki and Medzhitov, 2010), we speculated that *nuc1* may be relevant for innate immune evasion instead of adaptive immune evasion. This was consistent with published researches which demonstrated that *S. aureus* escapes innate immune defense through NET degradation and phagocyte apoptosis (Berends et al., 2010; Thammavongsa et al., 2013; Winstel et al., 2018). However, concerning *nuc2*, no observable immune evasion function was detected in our study. It may be due to its reported low enzymic activity (Kiedrowski et al., 2014), and thus, NETs could not be sufficiently degraded with *nuc2*.

Considering patients with IAIs require long-term administration of antibiotics, our study also examined the impact of antibiotics on nuclease expression. Although MSSA and MRSA had different responses to antibiotics, ceftriaxone and daptomycin reduced *nuc1* levels in both MSSA and MRSA. Because MRSA strains are resistant to ceftriaxone, which seems to downregulate *nuc1* expression, the use of this antibiotic could lead to increased biofilm formation. As such, caution should be taken when adopting ceftriaxone for IAI treatment before the antibiotic sensitivity testing is clear.

Our study has some limitations. First, the higher biofilm-forming capacity of IAI groups cannot be fully explained by the low expression of thermonucleases, as other factors such as *sarA*, *clfA/B*, *srtA*, and *agr* loci also contribute to biofilm formation regulation (Paharik and Horswill, 2016; Otto, 2018). Second, the regulation mechanism of *nuc2* transcription in *S. aureus* remains unknown. Finally, in our hematogenous IAI mouse model, we did not check the bacterial load in other systemic organs, which could help us understand the capacity of these strains to disperse to distant sites. Also, the bacterial load in the hematogenous IAI mouse model showed no significant differences even in the Δ*nuc1/2* group. Although the mechanism underlying the discrepancy observed between the two mouse models remains unclear, we speculated that the virulence adopted by *S. aureus* to colonize bone implants and develop biofilms might vary depending on how they invade the human body.

In summary, we identified temporal regulation for *nuc1* and *nuc2*. The pathogenesis of both nucleases was explored using two types of infection models. Low expression of both *nuc1* and *nuc2* is essential in *S. aureus* IAIs caused by surgical site contamination. However, in hematogenous IAIs, upregulation of *nuc1*, rather than *nuc2*, contributes to *S. aureus* pathogenesis. Our study may provide new insights into the prevention and treatment of IAIs.

## Materials and Methods

### Bacterial Strains and Growth Conditions

*Staphylococcus aureus* strains used in this study were either strains that were maintained in our laboratory or clinically isolated. To construct a fluorescence-labeled strain, pRN11 and pCM29 plasmids (Pang et al., 2010; de Jong et al., 2017) expressing mCherry and sfGFP, respectively, were introduced into *S. aureus* competent cells RN4220 via electroporation using a MicroPulser (Bio-Rad, United States). After adding 0.5 μg of plasmid into 50 μl of RN4220 competent cells, the default Staph program was performed (2-mm gap, 1.8 kV, 2.5 ms). Then, the cells were immediately resuspended in 1 ml of TSB (Haibo, Qingdao, China) and cultured on a shaking incubator at 200 rpm for 1 h at 37°C. A total of 100 μl of the recovery culture was grown overnight at 37°C on a TSB-chloramphenicol (10 μg/ml) agar plate. Next morning, a single chloramphenicol-resistant colony harboring pRN11 or pCM29 plasmids was selected and grown overnight in 4 ml of TSB with chloramphenicol (10 μg/ml). Next, according to a previously described bacteriophage transformation method (Olson, 2014), the plasmid was transformed into *S. aureus* ST1792, which was isolated from an infected prosthesis, with bacteriophage 11.

### Thermonuclease Activity Detection

Bacterial cultures were grown in TSB on a shaking incubator at 37°C and 200 rpm for 6 h and then heat-inactivated at 100°C for 10 min. Toluidine blue DNase agar was used to detect thermonuclease activity according to the manufacturer’s instructions (Haibo, Qingdao, China). A total of 80 μl of inactivated culture was added into a 5-mm-diameter hole in the agar plate made with a sterile pellet tip. The plate was incubated for 6 h at 37°C, and the diameter of the clearing zone was measured.

### Construction of Thermonuclease Mutants

In-frame deletion of *nuc1* and *nuc2* genes in clinical isolate ST1792 was performed by allelic replacement using the plasmid pKOR1 as previously described elsewhere (Bae and Schneewind, 2006). The primers used are listed in Supplementary Table 1. Briefly, after amplifying the upstream and downstream regions of the target gene, we used SOE-PCR to ligate the upstream and downstream fragments. The PCR product was cloned into pKOR1, and the resulting recombinant plasmids pKOR1-*nuc1* and pKOR1-*nuc2* were further transformed into *S. aureus* competent cells RN4220 via electroporation and maintained using chloramphenicol (10 μg/ml). Next, the plasmid was transformed into *S. aureus* ST1792 using bacteriophage 11. *S. aureus* ST1792 containing the plasmid constructed was used for construction of mutants by allele replacement with temperature shifting as described previously (Bae and Schneewind, 2006). Candidate mutant strains were validated by Sanger sequencing.

### *In vitro* Static Biofilm Assays

All bacterial strains involved in this experiment were cultured overnight at 37°C in TSB supplemented with 0.25% glucose (TSBG). The overnight culture was serially diluted to a concentration of ∼1 × 10^6^ CFU/ml, and then, 100 μl of the culture was inoculated into a 96-well plate with a flat bottom (BIOFIL, Guangzhou, China). UHMWPE disks (5-mm diameter) were sterilized and placed on a 96-well plate with a round bottom (BIOFIL) and then inoculated with 100 μl of the bacterial culture followed by incubation. Titanium disks (10-mm diameter) were sterilized and placed on a 24-well plate with a flat bottom (BIOFIL) and then inoculated with 1 ml of bacterial culture followed by incubation. After incubation at 37°C for 24 h, the culture medium was aspirated from each well, and wells were washed three times with either 200 μl of PBS in case of the 96-well plate or 1 ml of PBS in case of the 24-well plate. After fixation with methanol, the plate was air-dried, and the biofilm was stained with 200 μl of crystal violet. The crystal violet bound at the bottom of the well was dissolved in 200 μl of 33% acetic acid, and 100 μl aliquots from each well were transferred into a new 96-well plate with a flat bottom. Optical absorbance was measured at 590 nm using a microplate reader (BioTek Instruments, Inc., United States) to quantify the biofilm biomass.

### Biofilm eDNA Content Measurement

*Staphylococcus aureus* biofilms were grown as described above in a six-well plate (1.5 ml TSBG per well). After gently removing the supernatant, biofilm cells were resuspended with 1 ml of PBS and then filtered using a 0.2-μm filter. To measure eDNA content, 100 μl of filtered resuspension was mixed with 100 μl of 2 μM SYTOX Green (Invitrogen, United States). Fluorescence was measured by using a plate reader (BIO-TEK, ELX 800, United States) with excitation and emission wavelengths of 485 and 520 nm.

### *Staphylococcus aureus* RNA Isolation and Quantitative PCR

To investigate the transcription levels of thermonucleases between an IAI strain and a non-IAI strain, *S. aureus* was cultured in TSB (or TSB supplied with 20% human synovial fluid) in a shaking incubator at 37°C and 200 rpm for 6 h. To investigate thermonuclease expression at different time points, ST1792 was cultured in TSB (37°C/200 rpm) and collected at several time points (1, 2, 4, 6, and 8 h). To determine *nuc1/2* expression in ST1792 and its isogenic mutants (Δ*nuc1* and Δ*nuc2*), bacteria were cultured in TSB and incubated at 37°C and 200 rpm for 6 h. To explore *nuc1/nuc2* transcription changes after exposure to antibiotic, ST1792 and USA300 were cultured in TSB supplied with sub-MIC antibiotics for 6 h (37°C/200 rpm). The abovementioned bacteria were harvested and transferred to a tissue lyser (Scientz^TM^, Ningbo, China), and the cell wall was physically disrupted for 30 s at a frequency of 50 Hz. RNA was isolated using the EZ-press RNA Purification Kit (EZBioscience, United States) according to the manufacturer’s instructions. The quality of the RNA was measured using a Nanodrop device (Thermo Fisher Scientific, United States), and RNA samples with absorbance ratios of 260 nm/280 nm and 260 nm/230 nm higher than 2.0 were selected for reverse transcription. After adding DNase to remove gDNA, 1 μg fresh RNA was immediately reverse-transcribed into cDNA using an RT-PCR kit (EZBioscience, United States). cDNA was diluted with ddH_2_O (1:5 dilution) and subsequently used as the DNA template for qPCR, performed with the kit 2 × SYBR Green qPCR Master Mix (EZBioscience). DNA amplification was performed by thermal cycling: initial denaturation at 95°C for 5 min, followed by 40 amplification cycles at 95°C for 10 s and at 60°C for 30 s using a Roche LightCycler 480 (Roche, Switzerland). The primers used in this study and other related information such as product size, primer efficiency, and cycling parameters are provided in Supplementary Table 1. Relative gene expression levels were quantified using the 2^–ΔΔ*CT*^ method, with the expression levels of *gyrB* as the internal reference.

### Biofilm Structure Observation Using SEM and Confocal Microscopy

Overnight-grown ST1792 biofilms were formed on a titanium disk as described above. After gently being washed with PBS, the samples were fixed with 2.5% glutaraldehyde at 4°C for 4 h, dehydrated using a graded ethanol series (50, 70, 80, 90, 95, and 100% v/v) for 10 min, freeze-dried, coated with platinum, and visualized using SEM (Magellan 400, FEI, United States).

For confocal microscopy observation, biofilms grown on titanium disk were stained with a Live/Dead BacLight bacterial viability kit (Invitrogen, United States) according to the instructions of the manufacturer. Stained biofilms were observed immediately using a confocal microscope (Leica TCS SP8, Germany). The optimal exposure time and laser intensity for both channels (excitation: 488 and 555 nm) were manually set to ensure no overexposure among groups. Then, all of the images were acquired with the same setting for comparability among groups. Raw data were imported into Imaris 9.0.1 for biovolume and biofilm thickness calculation.

### Detection of Bacterial Clumps Through Flow Cytometry

sfGFP-labeled ST1792 and its isogenic mutants were incubated in TSB at 37°C and 200 rpm overnight in the presence of chloramphenicol. Then, overnight bacterial cultures were used for flow cytometry (Beckman Coulter CytoFLEX, United States). The sample flow rate was 10 μl/min, and 20,000 events were recorded for further analysis. First, the GFP+ cell population, comprising the bacteria, was selected. Then, single/clumping populations were labeled using the correlation between FITC-H and FITC-A.

### Neutrophil Extracellular Traps Degradation Assay

Neutrophils were isolated from the blood of healthy donors. The anti-coagulated whole blood (5 ml) was carefully layered over 5.0 ml of Polymorphprep^TM^ (Alere Technologies, Norway) in a 15-ml centrifuge tube. Tubes were centrifuged at 500× *g* for 30 min at 18–22°C. PMNs were gently separated, and 3 ml of RPMI medium was added to restore normal osmolality. Samples were centrifuged at 400× *g* for 10 min to collect cells. Finally, the cells were resuspended in the RPMI medium containing 10% fetal bovine serum (FBS). For NET induction, phorbol-12-myristate-13-acetate (PMA, Sigma, United States) was added to the culture medium to reach a final concentration of 90 nM, and samples were incubated at 37°C with 5% CO_2_ for 4 h. Then, heat-inactivated bacterial culture (ST1792 and its isogenic mutants) was added (MOI = 100) and incubated for 2 h to degrade the NETs. After fixation with 4% paraformaldehyde, NETs were stained with SYTOX Green (Invitrogen, United States) and observed using a Leica DMI8 microscope (Leica, Germany).

### Cellular Cytotoxicity Assays Using Trypan Blue Staining

Human monocytic THP-1 cells were obtained from the Institute of Biochemistry and Cell Biology (Shanghai, China). A total of 1 × 10^5^ THP-1 cells were cultured, overnight, in an RPMI medium containing 10% FBS and penicillin/streptomycin together with heat-inactivated WT, Δ*nuc1*, Δ*nuc2*, or Δ*nuc1/2* bacterial suspensions (MOI = 10) and DNA (final concentration of 100 ng/μl). Subsequently, cells were stained with trypan blue and visualized using light microscopy.

### Bacterial MIC Assay

We adopted a macrodilution method to determine the MIC of different antibiotics for MRSA and MSSA, and 1 ml of twofold serial dilutions of antibiotics dissolved in TSB was added to 1 ml of TSB, which contained nearly 10^6^ CFU/ml, in separate tubes. After overnight incubation at 37°C, the turbidity of the test tubes was visually inspected, as turbid test tubes were indicative of bacterial growth, whereas tubes that remained clear indicated no growth. The MIC of the antibiotics tested was considered to be the lowest concentration that inhibited growth. Gentamycin, linezolid, daptomycin, ciprofloxacin, and ceftriaxone were purchased from Aladdin (Shanghai, China).

### GEO Microarray Data Analysis

Public microarray dataset GSE25454 including 74 samples was selected. First, we downloaded the raw data (.CEL files) from the GEO database (GSE25454). Then, the following process was performed in R (4.0.1). We used the readAffy function (limma package; Ritchie et al., 2015) to import the .CEL files and performed background correction with the gcrma function (GCRMA package; Lim et al., 2007). Note that the resulting expression data were represented as intensity and transformed into log-2 scale according to descriptions in the GCRMA package manual. After that, we used the “normalizeWithinArray” function (from the limma package; Ritchie et al., 2015) to normalize the data among samples to remove batch effects. Finally, we extracted the *nuc1* and *nuc2* expression values for each sample at various time points and performed a Pearson correlation test. The scatter plot was presented with the ggscatter function (ggpubr package).

### Implant-Associated Infections Mouse Model

#### Mice Model of Exogenous IAIs

Twenty BALB/c mice (6 weeks old) were randomly divided into four groups (WT, Δ*nuc1*, Δ*nuc2*, and Δ*nuc1/2*). Mice were intraperitoneally anesthetized with 1% pelltobarbitalum natricum (provided by the animal center), and both knees were shaved and disinfected. Then, the distal femur was exposed through a medial parapatellar incision, and a narrow channel was created at the femoral end using a 25G needle. Subsequently, the prepared sterile titanium wires (0.5-mm diameter) were inserted in a retrograde direction into the intramedullary canal. The overlying subcutaneous tissue and skin were closed using absorbable subcuticular sutures. Finally, 25 μl of the corresponding bacterial inoculum (ST1792 and its isogenic mutants, ∼1 × 10^7^/ml) was injected intraarticularly into the knee joint space. All mice were anesthetized and euthanized by cervical dislocation 7 days after the infection. Peri-implant tissues were harvested and homogenized in 1 ml of sterile saline before CFU counting. The biofilm on the titanium wires together with 1 ml of sterile saline was subjected to sonication (30 kHz, 10 min) in an ultrasound bath (CQ-200B-DST, Yuejin, China), and the resulting sonicated fluid was used for further bacterial load quantification.

#### Competition Infection Model

To observe the *in vivo* biofilm structure and explore the adaptability of different *S. aureus* genotypes, a titanium disk was implanted subcutaneously into the dorsal area of the mice, and ∼1 × 10^6^ CFU of the strain mixture was inoculated around the implant. We labeled Δ*nuc1*/WT with sfGFP and Δ*nuc2*/Δ*nuc1/2* with mCherry. Mice were infected with either a WT/Δ*nuc1/2* mixture or a Δ*nuc1*/Δ*nuc2* mixture. All mice were euthanized by cervical dislocation and anesthetized 7 days after the infection. The biofilm on the titanium disks was observed using a fluorescence microscope (Leica DMI8, Germany).

#### Mouse Model of Hematogenous IAIs

Thirty-two adult BALB/c mice were randomly divided into four groups (WT, Δ*nuc1*, Δ*nuc2*, and Δ*nuc1/2*). After the implantation surgery performed as described previously, mice were infected via tail vein injection (ST1792 and its isogenic mutants, 1 × 10^7^ CFU/100 μl). Survival was recorded daily. All mice were anesthetized and euthanized by cervical dislocation 28 days after the infection. Peri-implant tissues and biofilm cells on the implant were prepared as described above. Bacterial load enumeration was performed according to the protocol listed in Section “Bacterial Load Enumeration.”

#### Bacterial Load Enumeration

The bacterial suspension or sonicated fluid was serially diluted tenfold. A total of 100 μl of the diluted suspension was spread on sheep blood agar plates. After incubation at 37°C overnight, CFU counts were performed according to the National Standard of China GB/T 4789.2 protocol. The resulting bacterial load for peri-implant tissues was normalized to tissue weight, and all bacterial loads were presented on a log_10_ scale.

### Statistical Analysis

The GEO microarray was analyzed with R 4.0.1. The fluorescence distribution pattern in Figure 4 was quantified with a Pearson correlation test using Coloc 2, a program in ImageJ (1.53c, Fiji). The remaining data analysis was performed using GraphPad Prism 8.3.0. Statistical significance was indicated as a two-sided *p* < 0.05. Results are represented as mean ± SD unless stated otherwise.

## Data Availability Statement

The raw data supporting the conclusions of this article will be made available by the authors, without undue reservation.

## Ethics Statement

Neutrophils were isolated from the blood of healthy donors. Human synovial fluid was collected from osteoarthritis patients before they received intra-articular injection of hyaluronic acid. The procedures were approved by the Ethics Committee of the Shanghai Sixth Peoples Hospital. The handling of mice and related procedures in this study were approved by the Animal Care and Experiment Committee of the Medical College of Shanghai Jiao Tong University affiliated Sixth People’s Hospital.

## Author Contributions

JY contributed to the concept of the study and wrote the manuscript. JD and FZ contributed to the qPCR experiment. YM and JY performed *S. aureus* mutant construction. JT and MH collected clinical strains and performed antibiotic susceptibility testing. JY, FJ, and FZ performed the *in vivo* experiment. QW and PH contributed to data analysis and data interpretation. HS contributed to the study design, manuscript editing and revision. All authors contributed to the article and approved the submitted version.

## Conflict of Interest

The authors declare that the research was conducted in the absence of any commercial or financial relationships that could be construed as a potential conflict of interest.

## Supplementary Material

Supplementary Figure 1 Transcription levels of thermonucleases in clinical isolates cultured in TSB (supplied with 20% human synovial fluid). Expression of *nuc1* **(A)** and *nuc2* **(B)** in IAI and non-IAI isolates (*n* = 14/group) determined by qPCR. Statistical significance was calculated using two-tailed Student’s *t*-test; ^∗^*p* < 0.05; ^∗∗^*p* < 0.01 vs. non-IAI strains.

Supplementary Figure 2 Biofilm eDNA measurement for clinical isolates and its relation to *nuc1* or *nuc2* transcription. **(A)** Biofilm eDNA content in IAI and non-IAI biofilms was measured by with SYTOX green staining and presented as fluorescence signals (*n* = 14/group). Two-tailed Student’s *t*-test was adopted; ^∗^*p* < 0.05 vs. the non-IAI group. **(B)** Biofilm eDNA content was related to *nuc1* or *nuc2* expression using the Pearson correlation test.

Supplementary Figure 3 **(A)** Sanger sequence results of *nuc1* and *nuc2* mutations with alignment to WT ST1792. **(B)** ST1792 *nuc1* and *nuc2* double mutation leads to the sticky characteristic of the bacterial colony.

Supplementary Figure 4 Bacterial load enumeration for biofilms formed *in vitro* by ST1792 and its isogenic mutants. Statistical significance was calculated using ANOVA with Dunnett multiple column comparisons. *n* = 3/group. ^∗∗^*p* < 0.01 vs. WT.

Supplementary Figure 5 Fluorescence co-localization test using ImageJ. **(A)** Δ*nuc1* and Δ*nuc2* co-localization 2D intensity plot. **(B)** WT and Δ*nuc1/2* co-localization 2D intensity plot. A Pearson correlation test was performed in this study.

Supplementary Figure 6 Biofilms formed on titanium disk *in vitro* by ST1792 and its isogenic mutant strains observed using SEM with ×2,000 magnification. Scale bar=10 μm.

Supplementary Figure 7 Biofilms formed by ST1792 and its isogenic mutant strains observed using a confocal microscope. Green represents live cells, and red represents eDNA and dead cells. Scale bar=50 μm.

Supplementary Figure 8 Mutual comparison of survival curves among four groups. **(A)** Survival analysis between WT (*n* = 8) and Δ*nuc1* (*n* = 7); **(B)** survival analysis between WT (*n* = 8) and Δ*nuc2* (*n* = 8); **(C)** survival analysis between WT (*n* = 8) and Δ*nuc1/2* (*n* = 7); **(D)** survival analysis between Δ*nuc1* (*n* = 7) and Δ*nuc2* (*n* = 8); **(E)** survival analysis between Δ*nuc1/2* (*n* = 7) and Δ*nuc2* (*n* = 8). Statistical significance was analyzed with a log-rank (Mantel–Cox) test.

Supplementary Figure 9 Bacterial count for implant (right) and peri-implant tissues (left) in a hematogenous IAI mouse model (*n* = 8, 12, 6, and 16 h for WT, Δ*nuc1*, Δ*nuc2*, and Δ*nuc1/2*, respectively). Statistical significance was calculated using ANOVA with Dunnett multiple column comparisons.

## Abbreviations

MRSA/MSSA: methicillin-resistant/methicillin-sensitive *Staphylococcus aureus*
EPS: extracellular polymeric substances
SEM: scanning electronic microscope
IAIs: implant-associated infections
MIC: minimum inhibitory concentration
TSB: tryptic soy broth.
