# Supplementary figures and images for: Thermonucleases Contribute to Staphylococcus aureus Biofilm Formation in Implant-Associated Infections–A Redundant and Complementary Story
Source: Front Microbiol. 2021 Jun 24;12:687888. doi: 10.3389/fmicb.2021.687888 (PMC8266213; doi:10.3389/fmicb.2021.687888)

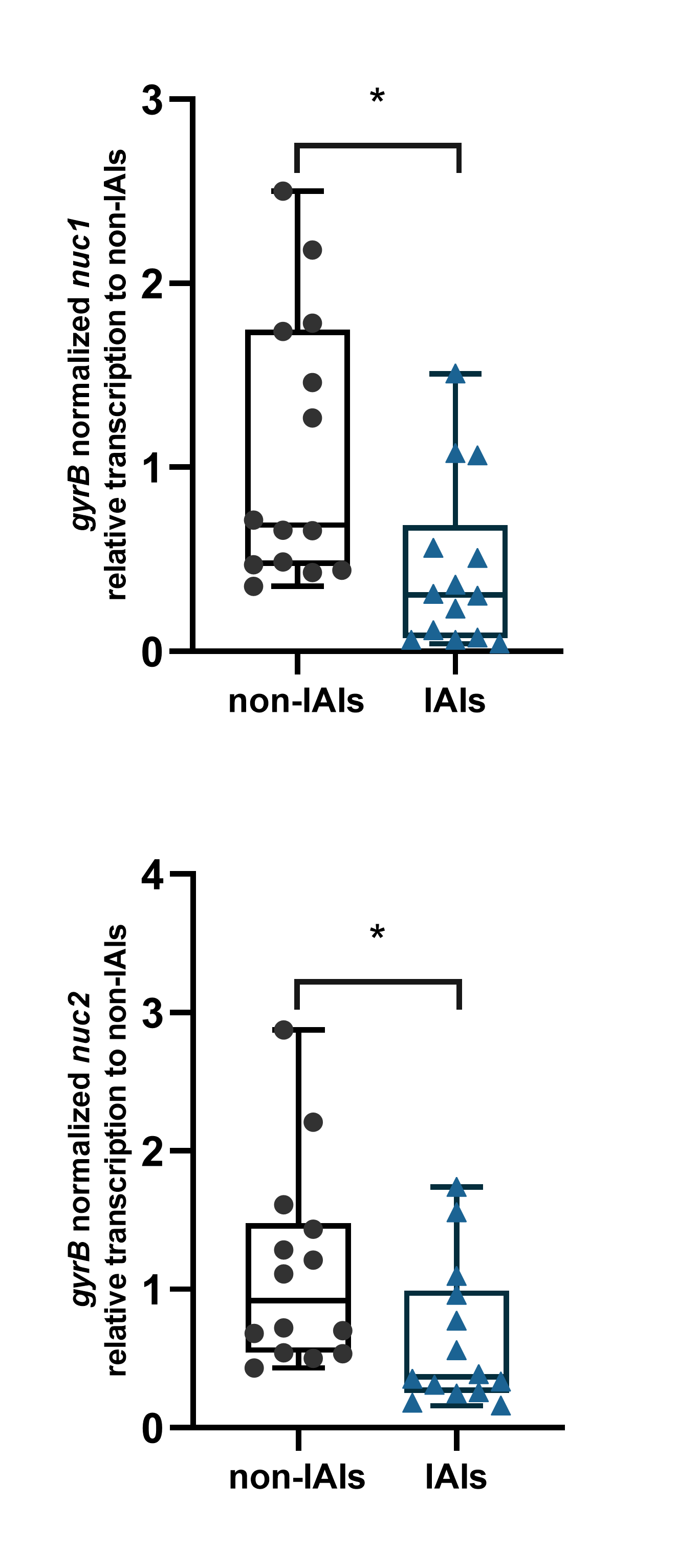

Supplement: Supplementary Figure 1 — Transcription levels of thermonucleases in clinical isolates cultured in TSB (supplied with 20% human synovial fluid). Expression of nuc1 (A) and nuc2 (B) in IAI and non-IAI isolates (n = 14/group) determined by qPCR. Statistical significance was calculated using two-tailed Student’s t-test; ∗p < 0.05; ∗∗p < 0.01 vs. non-IAI strains. [file Image_1.TIF]

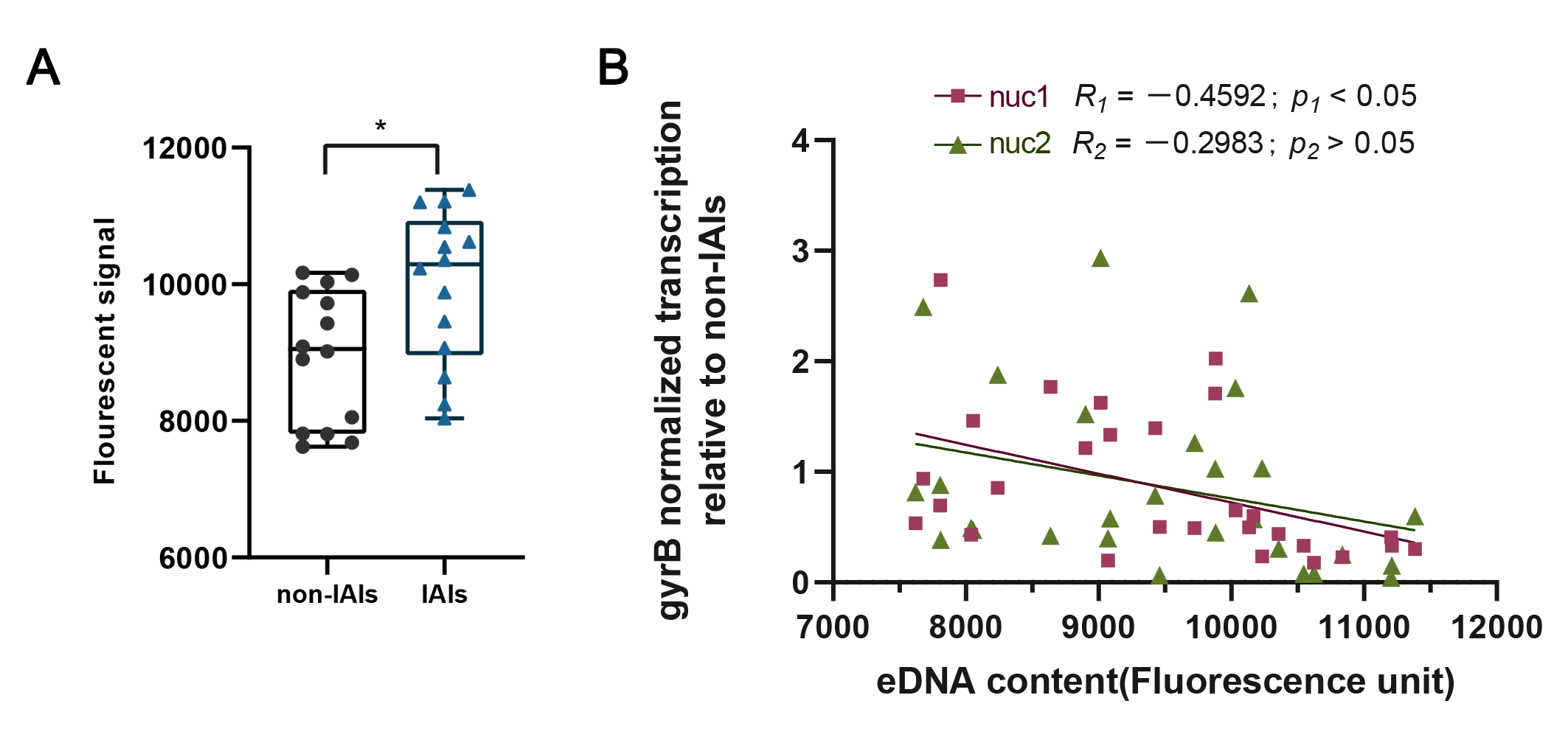

Supplement: Supplementary Figure 2 — Biofilm eDNA measurement for clinical isolates and its relation to nuc1 or nuc2 transcription. (A) Biofilm eDNA content in IAI and non-IAI biofilms was measured by with SYTOX green staining and presented as fluorescence signals (n = 14/group). Two-tailed Student’s t-test was adopted; ∗p < 0.05 vs. the non-IAI group. (B) Biofilm eDNA content was related to nuc1 or nuc2 expression using the Pearson correlation test. [file Image_2.TIF]

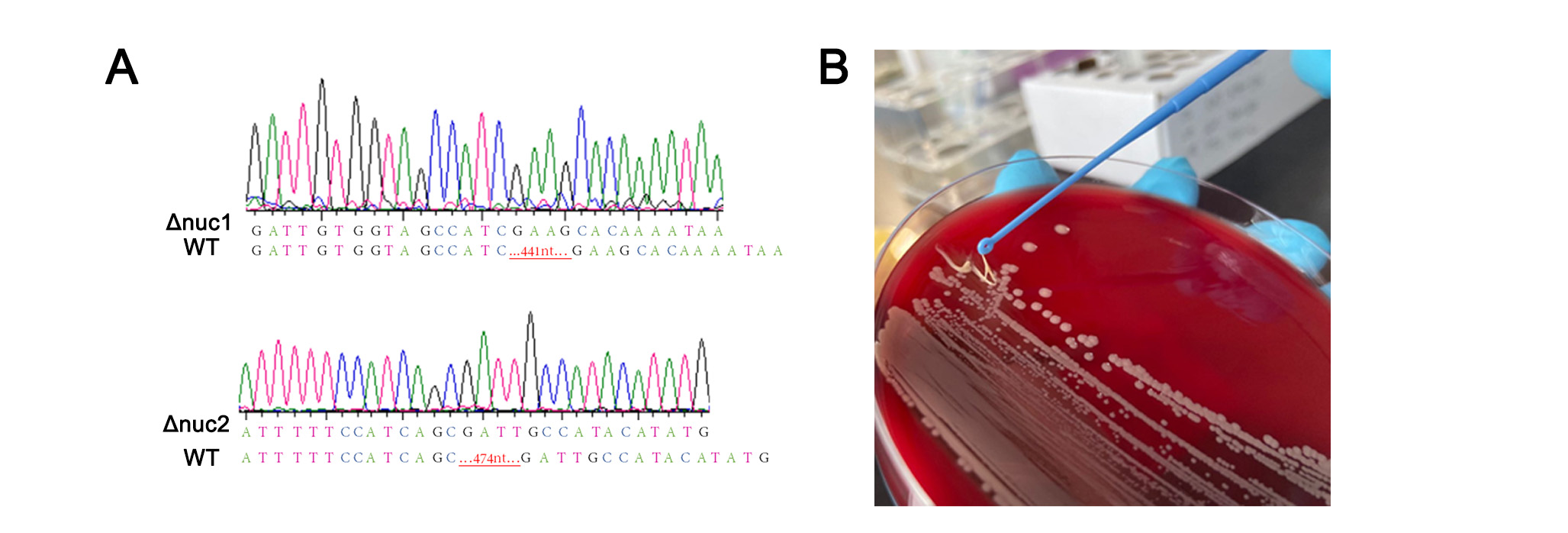

Supplement: Supplementary Figure 3 — (A) Sanger sequence results of nuc1 and nuc2 mutations with alignment to WT ST1792. (B) ST1792 nuc1 and nuc2 double mutation leads to the sticky characteristic of the bacterial colony. [file Image_3.JPEG]

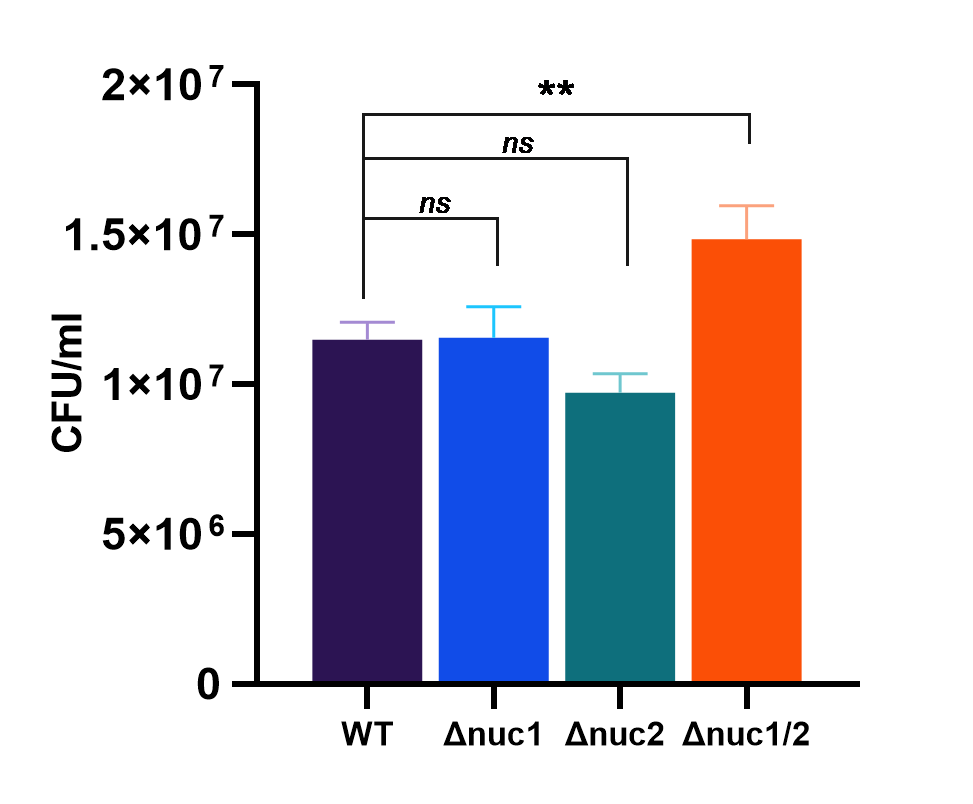

Supplement: Supplementary Figure 4 — Bacterial load enumeration for biofilms formed in vitro by ST1792 and its isogenic mutants. Statistical significance was calculated using ANOVA with Dunnett multiple column comparisons. n = 3/group. ∗∗p < 0.01 vs. WT. [file Image_4.TIF]

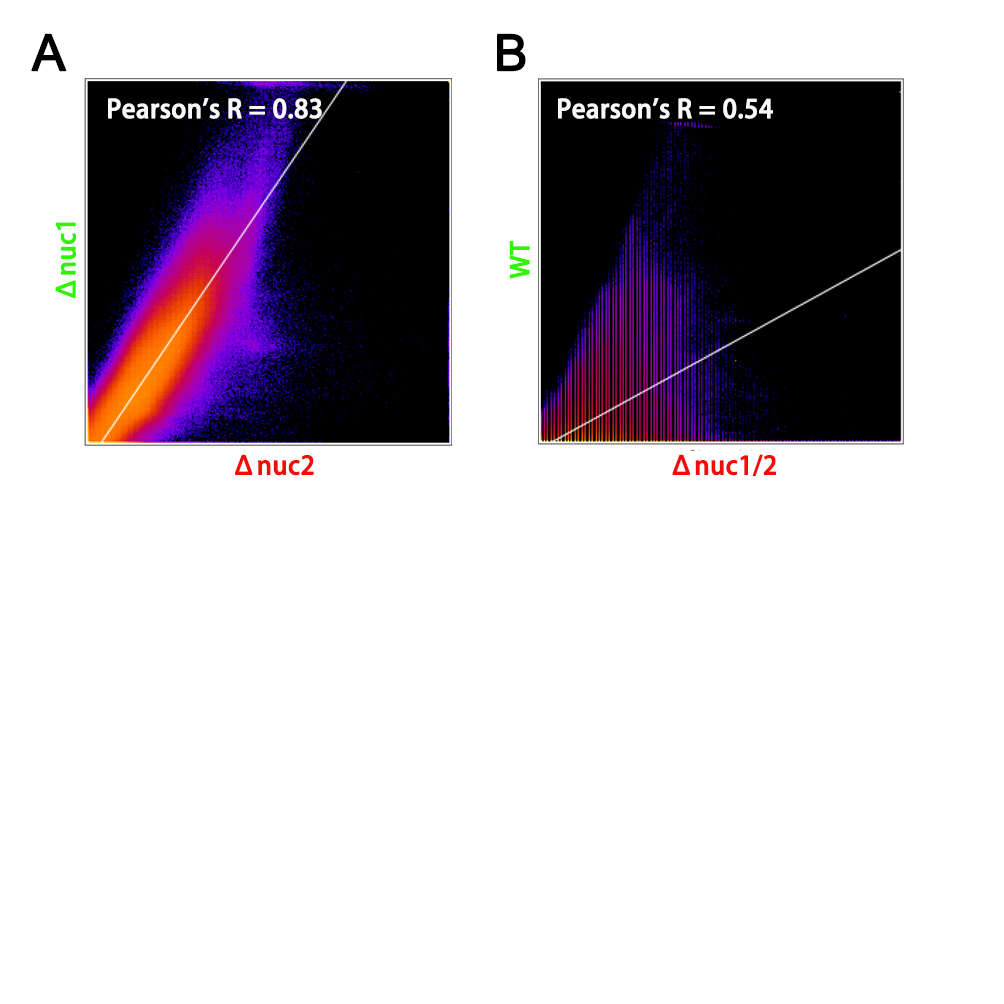

Supplement: Supplementary Figure 5 — Fluorescence co-localization test using ImageJ. (A) Δnuc1 and Δnuc2 co-localization 2D intensity plot. (B) WT and Δnuc1/2 co-localization 2D intensity plot. A Pearson correlation test was performed in this study. [file Image_5.TIF]

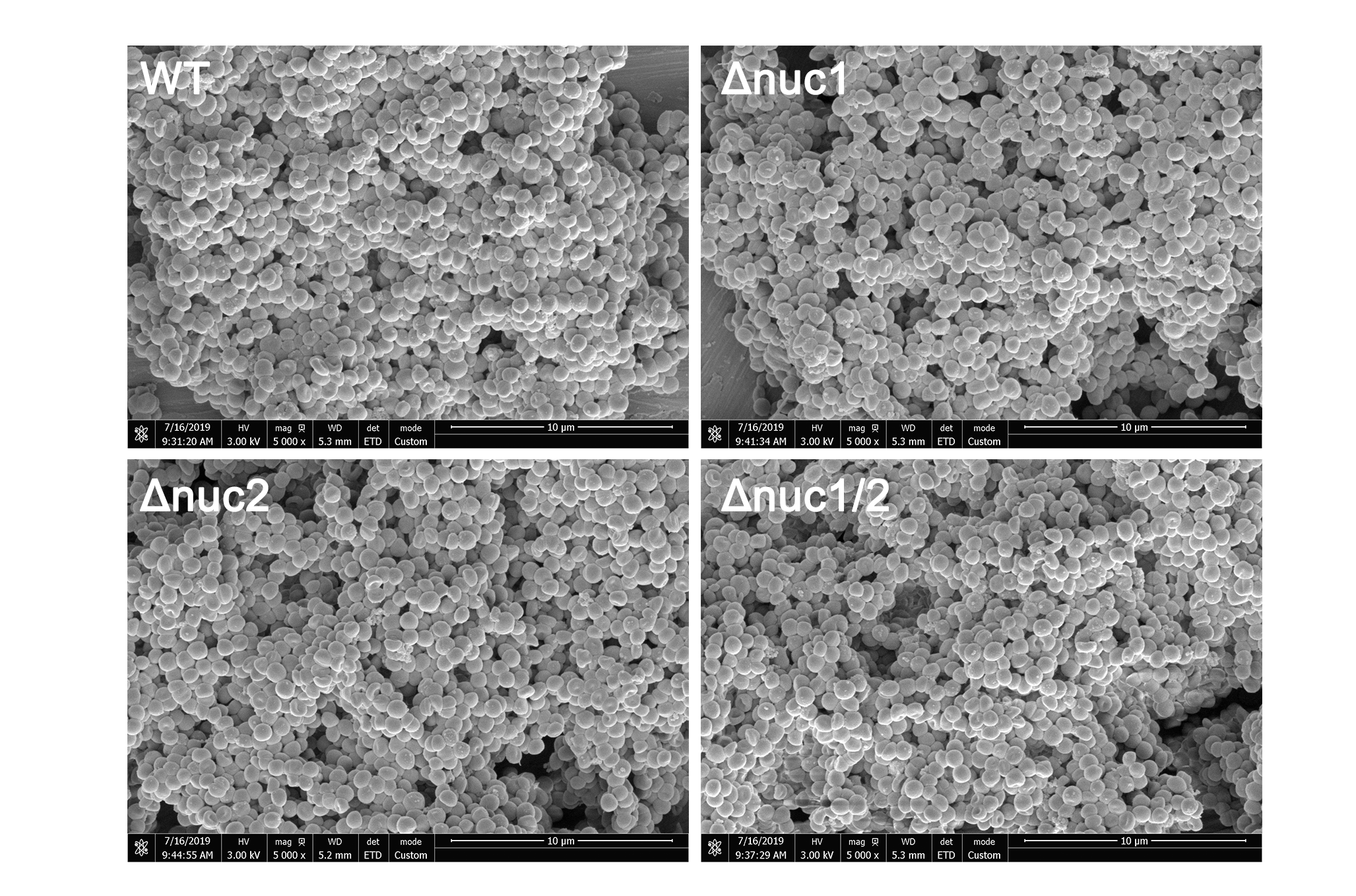

Supplement: Supplementary Figure 6 — Biofilms formed on titanium disk in vitro by ST1792 and its isogenic mutant strains observed using SEM with ×2,000 magnification. Scale bar=10 μm. [file Image_6.TIF]

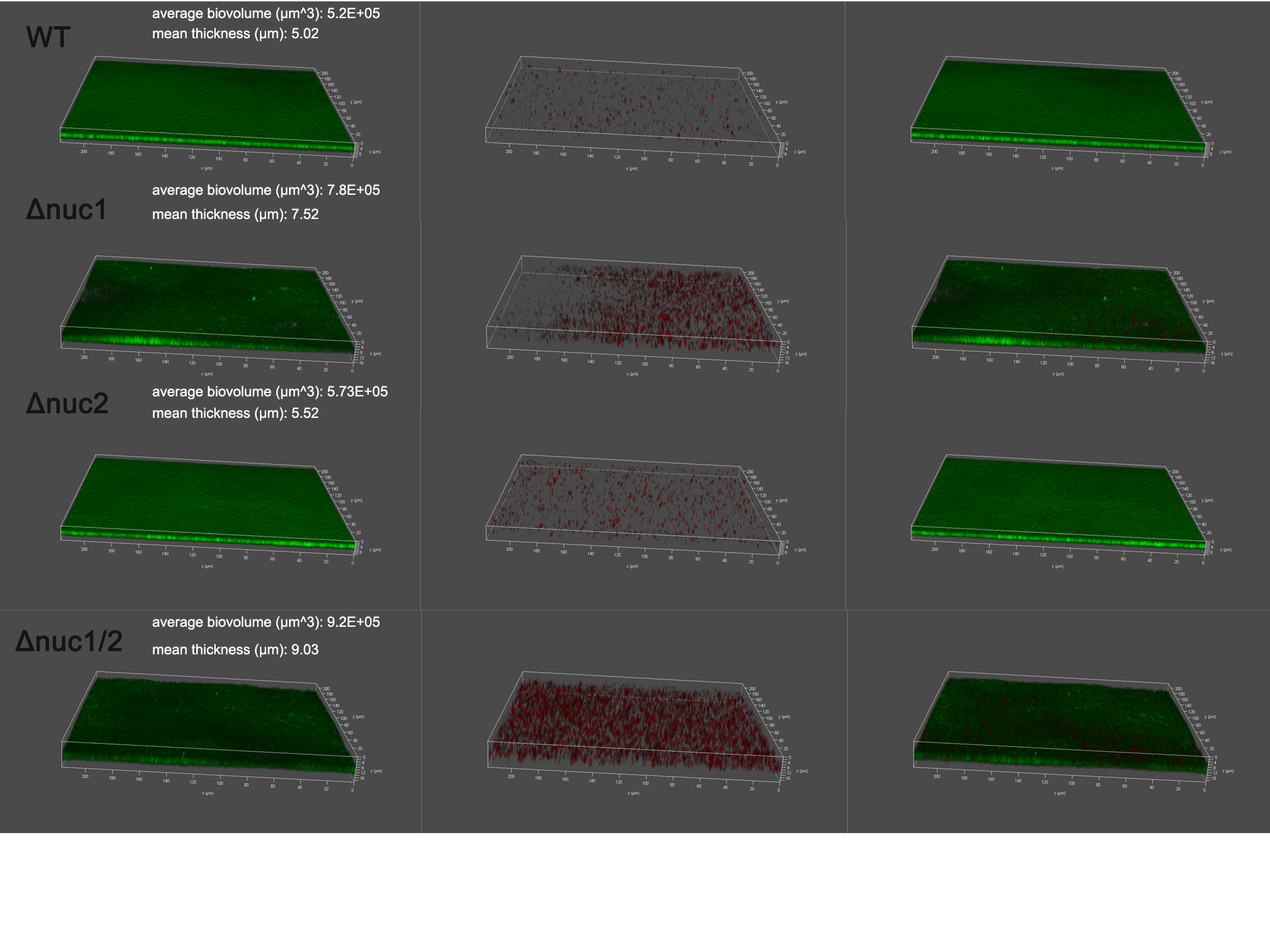

Supplement: Supplementary Figure 7 — Biofilms formed by ST1792 and its isogenic mutant strains observed using a confocal microscope. Green represents live cells, and red represents eDNA and dead cells. Scale bar=50 μm. [file Image_7.TIF]

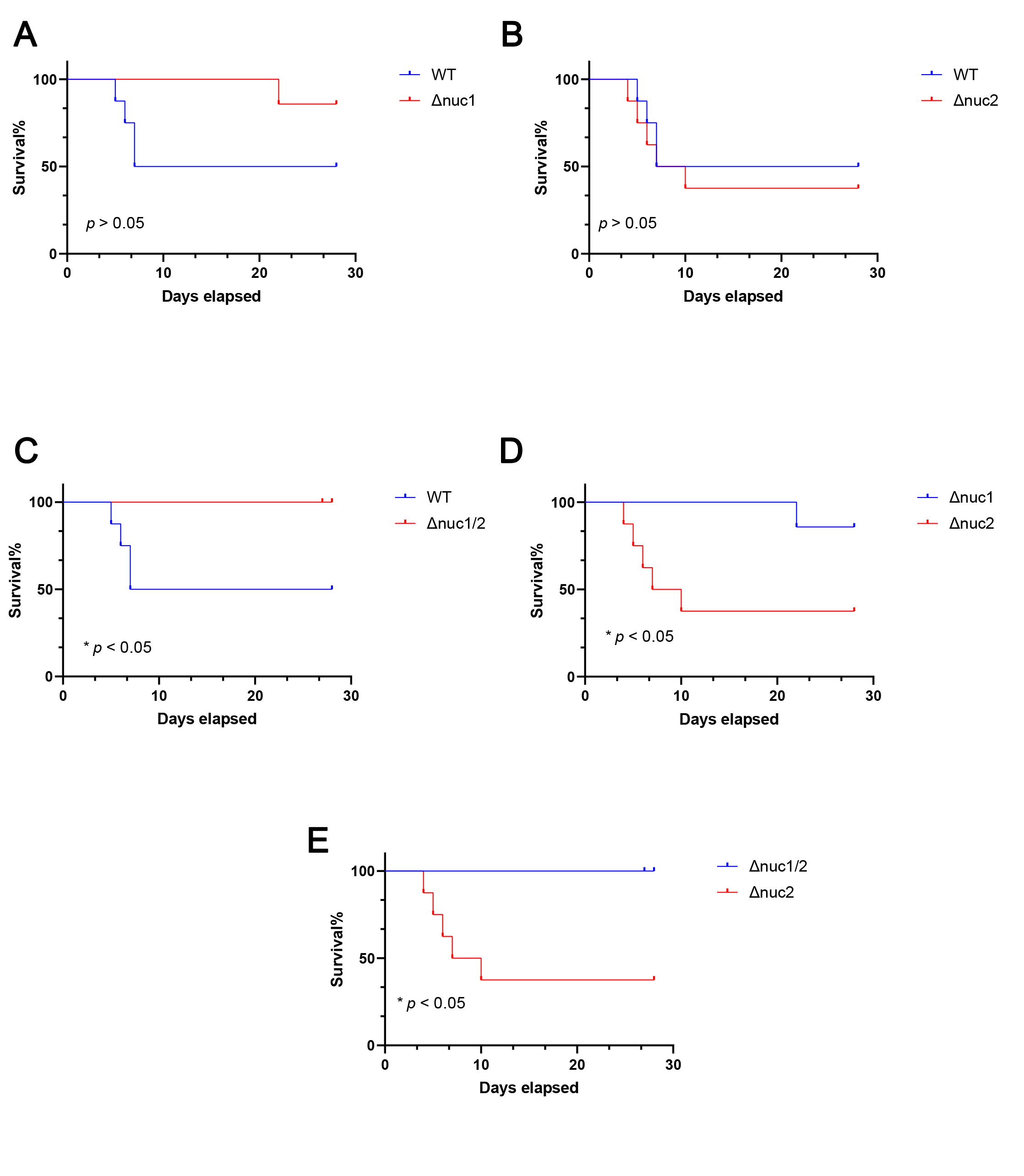

Supplement: Supplementary Figure 8 — Mutual comparison of survival curves among four groups. (A) Survival analysis between WT (n = 8) and Δnuc1 (n = 7); (B) survival analysis between WT (n = 8) and Δnuc2 (n = 8); (C) survival analysis between WT (n = 8) and Δnuc1/2 (n = 7); (D) survival analysis between Δnuc1 (n = 7) and Δnuc2 (n = 8); (E) survival analysis between Δnuc1/2 (n = 7) and Δnuc2 (n = 8). Statistical significance was analyzed with a log-rank (Mantel–Cox) test. [file Image_8.TIF]

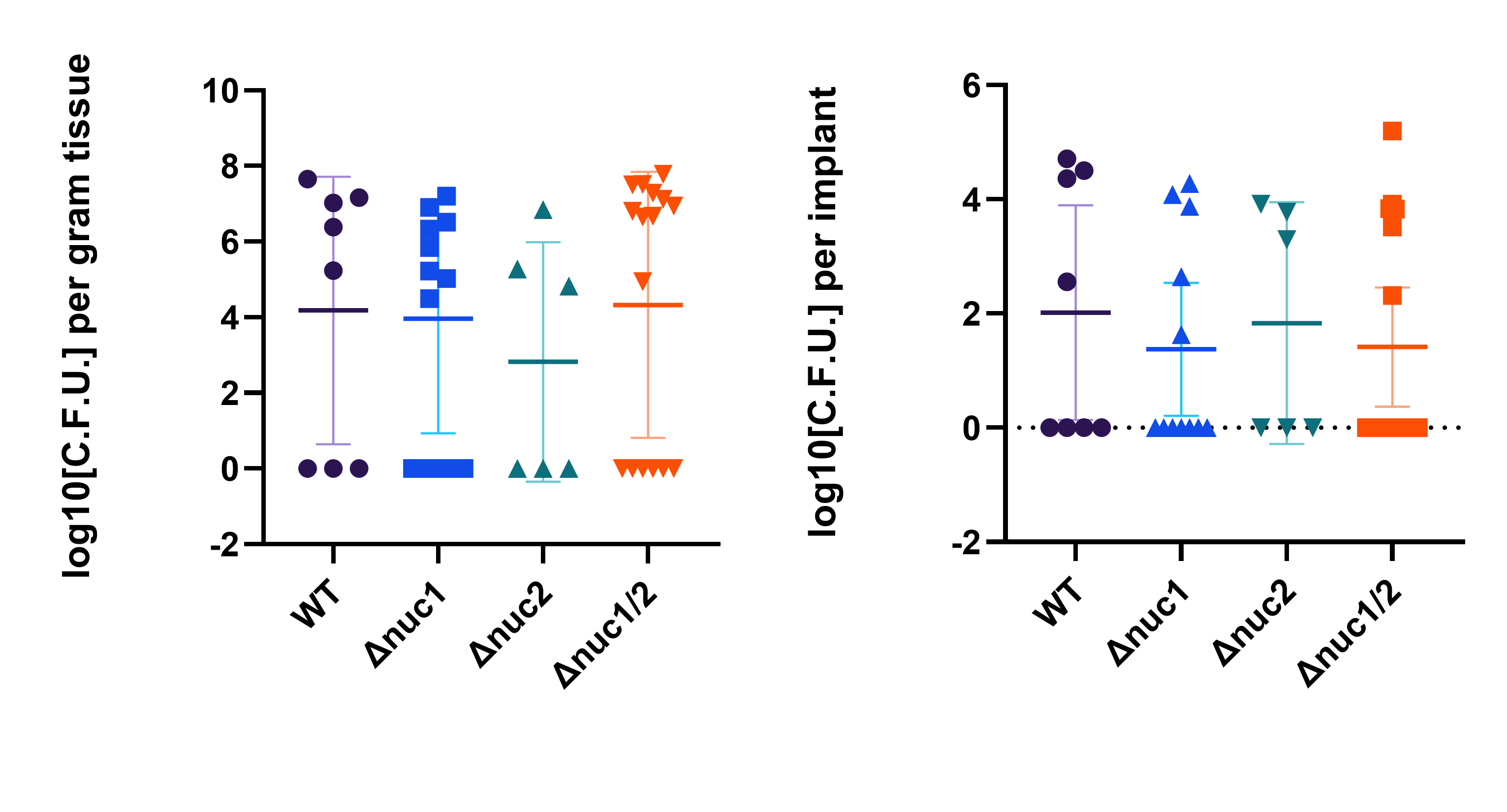

Supplement: Supplementary Figure 9 — Bacterial count for implant (right) and peri-implant tissues (left) in a hematogenous IAI mouse model (n = 8, 12, 6, and 16 h for WT, Δnuc1, Δnuc2, and Δnuc1/2, respectively). Statistical significance was calculated using ANOVA with Dunnett multiple column comparisons. [file Image_9.TIF]
